# Estimating uterine source current during contractions using magnetomyography measurements

**DOI:** 10.1371/journal.pone.0202184

**Published:** 2018-08-23

**Authors:** Mengxue Zhang, Patricio S. La Rosa, Hari Eswaran, Arye Nehorai

**Affiliations:** 1 Preston M. Green Department of Electrical and Systems Engineering, Washington University in Saint Louis, Saint Louis, Missouri, United States of America; 2 Geospatial Analytics, Global IT Analytics, Monsanto Company, Saint Louis, Missouri, United States of America; 3 Department of Obstetrics and Gynecology, University of Arkansas for Medical Sciences, Little Rock, Arkansas, United States of America; PreTel, UNITED STATES

## Abstract

Understanding the uterine source of the electrophysiological activity of contractions during pregnancy is of scientific interest and potential clinical applications. In this work, we propose a method to estimate uterine source currents from magnetomyography (MMG) temporal course measurements on the abdominal surface. In particular, we develop a linear forward model, based on the quasistatic Maxwell’s equations and a realistic four-compartment volume conductor, relating the magnetic fields to the source currents on the uterine surface through a lead-field matrix. To compute the lead-field matrix, we use a finite element method that considers the anisotropic property of the myometrium. We estimate the source currents by minimizing a constrained least-squares problem to solve the non-uniqueness issue of the inverse problem. Because we lack the ground truth of the source current, we propose to predict the intrauterine pressure from our estimated source currents by using an absolute-value-based method and compare the result with real abdominal deflection recorded during contractile activity. We test the feasibility of the lead-field matrix by displaying the lead fields that are generated by putative source currents at different locations in the myometrium: cervix and fundus, left and right, front and back. We then illustrate our method by using three synthetic MMG data sets, which are generated using our previously developed multiscale model of uterine contractions, and three real MMG data sets, one of which has simultaneous real abdominal deflection measurements. The numerical results demonstrate the ability of our method to capture the local contractile activity of human uterus during pregnancy. Moreover, the predicted intrauterine pressure is in fair agreement with the real abdominal deflection with respect to the timing of uterine contractions.

## Introduction

It is an inverse problem to estimate the underlying source currents, such as location and time courses, from electromagnetic measurements of uterine contractions. Solving this inverse problem is important for understanding the physiological, functional, and pathological properties of the uterus, which can be helpful in the diagnosis of labor and treatment of obstetric syndromes associated with contractile dysfunction such as preterm birth, post-term birth, and dysfunctional labor, to name a few. Uterine contractile dysfunction during pregnancy is a significant healthcare challenge that has long-term medical and financial consequences [1–3]. Therefore, investigating this inverse problem can lead to considerable clinical benefits for both mothers and children.

Currently, there is little work focusing on estimating the source currents of uterine activities during pregnancy [4, 5]. In [4], the authors investigated this problem based on simulated electrohysterogram data, also known as electromyography (EMG). However, EMG, which arises from the volume current and is recorded by electrodes attached to the abdomen, is strongly dependent upon tissue conductivity [6], resulting in severe attenuation when electrophysiological signals propagate to the abdominal surface. Unlike EMG, magnetomyography (MMG), without making electrical contact with the body and arising from the primary current, is much less dependent on tissue conductivity [7] and is independent of any kind of reference, ensuring the record of uterine localized activities. The authors in [5] evaluated the ability of a simulated full-coverage biomagnetic device to non-invasively monitor uterine magnetic activities. The device, however, is currently unavailable for taking the MMG measurements. A device, called SARA: SQUID (superconducting quantum interference device) array for reproductive assessment, is developed to non-invasively collect the abdominal MMG data of uterine contractions in [8].

In this work, derived from the quasistatic Maxwell’s equations, we develop a linear forward model of the abdominal magnetic field of uterine contractile events with respect to source current dipoles in an anisotropic myometrium. Based on this linear model, we conduct our primary estimation of source currents for both synthetic MMG data sets, generated using our multiscale model of uterine contractions [9], and real MMG data sets, collected using the SARA device. We also predict the corresponding intrauterine pressure from the estimated source currents in order to explore its clinical implications. To the best of our knowledge, our results are the first to estimate source currents in uterus during real contractions.

The mathematical notation used in this paper is as follows: Italic lowercase or uppercase letters denote scalars; bold italic lowercase letters indicate vectors; bold italic uppercase letters denote matrices, except for vector fields, which are in bold calligraphic uppercase e.g., electric field ***E***, magnetic field ***B***, current density ***J***, and lead field L. The *ℓ*_1_ and *ℓ*_2_ norms defined in the Euclidean space are denoted by ‖ ⋅ ‖_1_ and ‖ ⋅ ‖_2_, respectively.

## Materials and methods

In this section, we describe the collection of real and synthetic MMG data, and discuss the source current distribution of uterine contractions during pregnancy. We introduce a forward model for the magnetic field based on a lead-field matrix that is constructed on a realistic four-compartment volume conductor, and provide the estimation of the underlying source currents and the corresponding intrauterine pressure.

### Clinical site and MMG data

**Fig 1:**
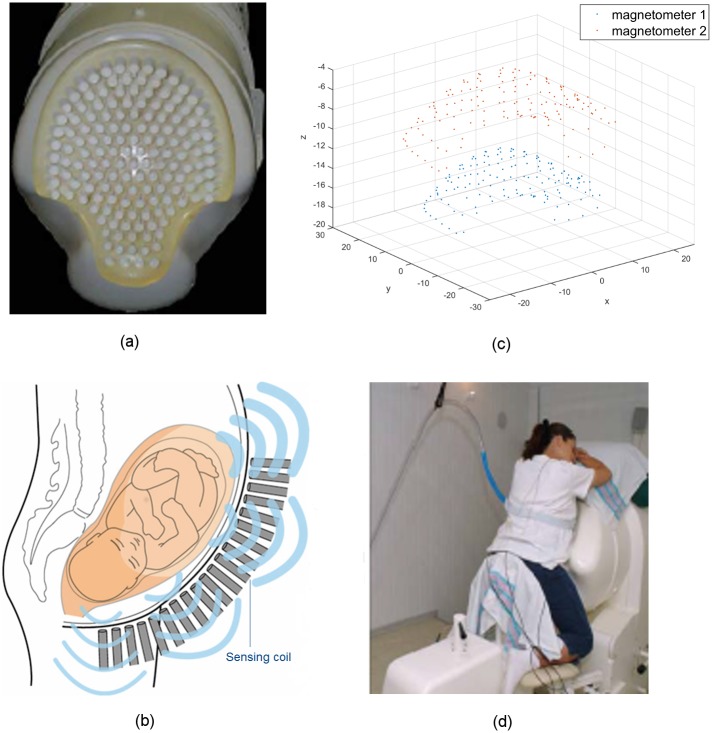
The SARA device used to non-invasively take MMG measurements of uterine activities. (a) The 151-channel sensor array, shown with the concave surface cover of the SARA device removed. (b) A simplified illustration of the sensing array and the uterine MMG field. (c) The layout of magnetometers of SQUID sensors (in centimeters). (d) Patient sits and leans against the surface of the array.

Three real MMG data sets were used for the estimation of uterine source current. These data sets were collected from two pregnant women at the University of Arkansas for Medical Sciences (UAMS), after the study protocol was explained and written consents to perform the study were obtained. The protocol was approved by the UAMS Institutional Review Board. The SARA device (Fig 1) we used to non-invasively collect the abdominal MMG data is installed in a magnetically shielded room next to the labor and delivery unit in the UAMS, to reduce external magnetic fields which interfere with the biomagnetic field generated by human organs. This SARA system consists of 151 primary magnetic sensors spaced 3 *cm* apart (Fig 1a), arranged in a concave array, covering the maternal abdomen from the pubic symphysis to the uterine fundus, and laterally over a similar span (Fig 1b). Each sensor measures the magnetic fields at two magnetometers, one of which is close to abdomen and the other one is 8 *cm* away from the first one in a direction similar but not identical to be normal to the SARA surface (Fig 1c), and the sensor measurement is the difference between the measurements from these two magnetometers. The difference between surface normal and the actual sensor orientation is particularly notable in areas such as the lower central portion of the SARA device due to space constraints in placing sensors in its convex surface. The patient simply sits and leans forward slightly against the smooth surface of the array (Fig 1d), allowing the SQUID sensors to receive electrophysiological signals.

All of the MMG data sets were first recorded at 250 Hz and then downsampled to 32 Hz. For the preprocessing, we applied a 8th-order band-pass Butterworth filter of 0.1 − 1 Hz to attenuate interfering maternal and fetal cardiac signals. A 8th-order band-stop (notch) Butterworth filter of 0.25 − 0.35 Hz was also applied to suppress maternal breathing, which is a prominent signal around 0.33 Hz. Noisy sensors were then removed to avoid possible pollution for MMG measurements. Among these data sets, one set has simultaneous recordings of the abdominal deflection, which were collected using an air-filled bag that was placed between the maternal abdomen and the SARA system. During uterine contractions, the pressure on the airbag induced by the abdominal shape change was transmitted via a tube to a pressure sensor that was connected to a standard fetal monitor which was located outside the shielded room. The output of the monitor was digitized and synchronized with the MMG signals. This simultaneous recording was performed as a proof of concept study and is difficult for routine application since noise artifacts could be introduced in the MMG data due to application of an external device.

**Fig 2:**
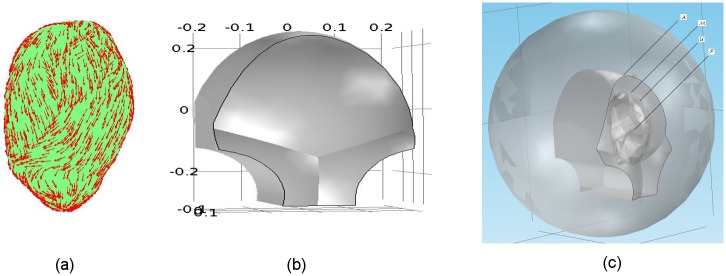
The anatomically realistic volume conductor. (a) Uterus, with fiber orientations displayed in red arrows. (b) Outer surface of abdomen conforming to the SARA device contour (in meters). (c) Four compartments of the volume conductor (from the outer layer to the inner layer): the abdominal cavity, A; the myometrium, M; the amniotic fluid, U; and the fetus, F.

Synthetic MMG data sets were employed to test our estimation approach. The synthetic MMG data sets were generated using our realistic multiscale electromagnetic model which was proposed in [9]. In this model, the volume conductor was exactly the same as the one in this inverse estimation work, and a sensor model was used to replicate the true SARA sensor positions and sensing directions as illustrated in Fig 1c. In particular, we represent the volume conductor (Fig 2) as four compartments (from the inner layer to the outer layer: fetus, amniotic fluid, uterus, and abdomen) with electrically conductive boundaries between compartments (Fig 2c). The geometry of an anatomically realistic uterus (Fig 2a) is based on the magnetic resonance images (MRI) of a pregnant, near-term woman and a uterine mesh is adopted from the FEMONUM project [10]. Considering the anisotropic nature and fiber variations of the myometrium, we divide the entire uterus into 25 contiguous regions. We set the region centers via random sampling from the finite-element mesh of uterus and then divide the regions by resampling any point that lies less than 4 *cm* from its nearest center. For each region, the fiber orientation, with respect to uterine surface tangential vector, is sampled from a normal distribution N∼(0,π/4) (see red arrows in Fig 2a for detailed fiber orientations). Assuming the cylindrical symmetry of fibers, the longitudinal and transversal conductivity values for the myometrium are 0.68 S/m and 0.22 S/m, respectively. We also assume that the abdomen (Fig 2b) deforms to follow the shape of the SARA device when patients lean against it to take the MMG measurement. The corresponding conductivity values for the abdomen, amniotic fluid, and fetus are 0.2 S/m, 1.74 S/m, and 0.5 S/m, respectively (see more details in our previous work [9]).

In the uterus, the myometrial cells either can spontaneously generate their own impulses, pacemaker cells, or can be excited by the action potentials propagating from the neighboring cells, pacefollower cells. However, it is still unclear whether there exists a specific pacemaker mechanism or a specific pacemaker area [11]. Except for some observations of contractile activity originating from specialized cells [12], most activities arise from any site throughout the myometrium [13, 14]. Among these sites, fundus is one possibility [15–17], hence we set the initiation of electrical activity to occur at the fundus in the simulations. This is an example location of the initiation area where the activity is excited, which makes no difference in our following analysis on source current. The final synthetic MMG data was generated by adding white noise with 5 fV/*√*Hz to the original synthetic time courses.

### Generation of electromagnetic fields

The electromagnetic fields of uterine contractions can be derived from the quasistatic approximation of Maxwell’s equations, since the frequency of the associated bioelectrical phenomena is typically below 1 kHz. Thus the time derivatives of the electromagnetic fields can be ignored as source terms. In the quasistatic approximation,

$$\begin{array}{c} \nabla \times E=0, \end{array}$$ (1)

$$\begin{array}{c} \nabla \cdot E=\frac{\rho }{\epsilon_{0}}, \end{array}$$ (2)

$$\begin{array}{c} \nabla \times B=\mu_{0}J, \end{array}$$ (3)

$$\begin{array}{c} \nabla \cdot B=0, \end{array}$$ (4)

where ***E*** and ***B*** are the electric and magnetic fields, respectively; *ϵ*_0_ and *μ*_0_ denote the permittivity and permeability of the free space, respectively; and *ρ* and ***J*** are the total charge density and current density, respectively. We divide the total current density, ***J***(***r***), into two components: the volume current, ***J***^v^ (***r***) = *σ* (***r***)***E***(***r***), which is the result of the macroscopic electric field in the volume conductor, and the primary current, ***J***^p^ (***r***):

$$\begin{array}{c} \begin{array}{cc} J(r) & =J^{p}\left(r\right)+J^{v}\left(r\right)\\ & =J^{p}\left(r\right)+\sigma \left(r\right)E\left(r\right), \end{array} \end{array}$$ (5)

where *σ*(***r***) is the macroscopic conductivity of the volume conductor. The primary current, ***J***^p^ (***r***), is related to the original biological activity, which is the source current density we consider in this work. From Eq (1), the electric field, ***E***, can be represented as the negative gradient of a scalar electrical potential, *V*, as

$$\begin{array}{c} E=-\nabla V. \end{array}$$ (6)

Therefore, the total current density in Eq (5) becomes

$$\begin{array}{c} J\left(r\right)=J^{p}\left(r\right)-\sigma \left(r\right)\nabla V\left(r\right). \end{array}$$ (7)

From Eqs (3) and (7), we obtain that ∇ · (∇ × ***B***) = 0 = *μ*_0_∇ · ***J*** = *μ*_0_∇ · (***J***^p^ − *σ*∇*V*), hence

$$\begin{array}{c} \nabla \cdot \left(\sigma \nabla V\right)=\nabla \cdot J^{p}, \end{array}$$ (8)

which shows that the scalar electrical potential, *V*, can be solved analytically using finite element method (FEM) [18] given the source current density, ***J***^p^, and proper boundary conditions.

### Linear forward model

The forward problem in uterine contractions is to calculate the magnetic field, ***B***(***r***), outside the abdomen generated by the source current density, ***J***^p^ (***r***), within the uterus. According to the Biot-Savart law, the magnetic field can be computed as

$$\begin{array}{c} B\left(r\right)=\frac{\mu_{0}}{4\pi }\int \frac{J(r^{\prime })\times l}{l^{3}}\;dv^{\prime }, \end{array}$$ (9)

where ***l*** = ***r*** − ***r***′ is the vector pointing from the source point ***r***′ to the observation point ***r*** with magnitude *l* = ‖***l***‖_2_. Here, the prime refers to quantities in the source region. Since ***l***/*l*^3^ = −∇(1/*l*) = ∇′(1/*l*), Eq (9) becomes

$$\begin{array}{c} \begin{array}{cc} B(r) & =\frac{\mu_{0}}{4\pi }\int J\left(r^{\prime }\right)\times \nabla^{\prime }\frac{1}{l}\;dv^{\prime }\\ & =\frac{\mu_{0}}{4\pi }\int (\frac{\nabla^{\prime }\times J\left(r^{\prime }\right)}{l}-\nabla^{\prime }\times \frac{J(r^{\prime })}{l})\;dv^{\prime }\\ & =\frac{\mu_{0}}{4\pi }\int \frac{\nabla^{\prime }\times J\left(r^{\prime }\right)}{l}\;dv^{\prime }-\frac{\mu_{0}}{4\pi }\int \nabla^{\prime }\times \frac{J(r^{\prime })}{l}\;dv^{\prime }\\ & =\frac{\mu_{0}}{4\pi }\int \frac{\nabla^{\prime }\times J\left(r^{\prime }\right)}{l}\;dv^{\prime }-\frac{\mu_{0}}{4\pi }\int {\hat{n}}^{\prime }\times \frac{J(r^{\prime })}{l}\;ds^{\prime }, \end{array} \end{array}$$ (10)

where ${\hat{n}}^{\prime }$ is the unit normal vector pointing outwards the source surface. For the total current density that approaches zero sufficiently fast when the source point, ***r***′, goes to infinity, Eq (10) becomes

$$\begin{array}{c} B\left(r\right)=\frac{\mu_{0}}{4\pi }\int \frac{\nabla^{\prime }\times J\left(r^{\prime }\right)}{l}\;dv^{\prime }. \end{array}$$ (11)

With Eq (7),

$$\begin{array}{c} \begin{array}{cc} B(r) & =\frac{\mu_{0}}{4\pi }\int \frac{\nabla^{\prime }\times \left(J^{p}\left(r^{\prime }\right)-\sigma \left(r^{\prime }\right)\nabla^{\prime }V\left(r^{\prime }\right)\right)}{l}\;dv^{\prime }\\ & =\frac{\mu_{0}}{4\pi }\int (\frac{\nabla^{\prime }\times J^{p}\left(r^{\prime }\right)}{l}-\frac{\nabla^{\prime }\times (\sigma \left(r^{\prime }\right)\nabla^{\prime }V\left(r^{\prime }\right))}{l})\;dv^{\prime }. \end{array} \end{array}$$ (12)

Since ∇ × (*σ*∇*V*) = ∇*σ* × ∇*V* + *σ*∇ × ∇*V* = ∇*σ* × ∇*V*,

$$\begin{array}{c} B\left(r\right)=\frac{\mu_{0}}{4\pi }\int (\frac{\nabla^{\prime }\times J^{p}\left(r^{\prime }\right)}{l}-\frac{\nabla^{\prime }\sigma \left(r^{\prime }\right)\times \nabla^{\prime }V\left(r^{\prime }\right)}{l})\;dv^{\prime }. \end{array}$$ (13)

With ∇*σ* × ∇*V* = *V*(∇ × ∇*σ*) − ∇ × (*V*∇*σ*) = −∇ × (*V*∇*σ*), the magnetic field, ***B***, is represented as

$$\begin{array}{c} \begin{array}{cc} B(r) & =\frac{\mu_{0}}{4\pi }\int (\frac{\nabla^{\prime }\times J^{p}\left(r^{\prime }\right)}{l}+\frac{\nabla^{\prime }\times \left(V\left(r^{\prime }\right)\nabla^{\prime }\sigma \left(r^{\prime }\right)\right)}{l})\;dv^{\prime }\\ & =\frac{\mu_{0}}{4\pi }\int \frac{\nabla^{\prime }\times \left(J^{p}\left(r^{\prime }\right)+V\left(r^{\prime }\right)\nabla^{\prime }\sigma \left(r^{\prime }\right)\right)}{l}\;dv^{\prime }. \end{array} \end{array}$$ (14)

Based on the equivalence between Eqs (9) and (11), we obtain from Eq (14) that

$$\begin{array}{c} B\left(r\right)=\frac{\mu_{0}}{4\pi }\int \left(J^{p}\left(r^{\prime }\right)+V\left(r^{\prime }\right)\nabla^{\prime }\sigma \left(r^{\prime }\right)\right)\times \frac{l}{l^{3}}\;dv^{\prime }. \end{array}$$ (15)

According to Eq (8), the electrical potential, *V*, in the above equation can be computed from the source current density, ***J***^p^, and is linearly related to ***J***^p^. Therefore, based on Eq (15), the magnetic field, ***B***, is linearly related to the source current density, ***J***^p^ [19]. Thus, there is a lead field, $L(r,r^{\prime })$, relating the magnetic field measurement, ***B***, at *r* to the source current, ***J***^p^, at ***r***′, satisfying

$$\begin{array}{c} B\left(r\right)=\int L\left(r,r^{\prime }\right)\cdot J^{p}\left(r^{\prime }\right)\;dv^{\prime }. \end{array}$$ (16)

If the source current, ***J***^p^, is a current dipole with moment ***q*** = *q****d***_q_ in location ***r***_q_, i.e., ***J***^p^(**r**) = ***q****δ*(***r*** − ***r***_q_), the magnetic field is given by

$$\begin{array}{c} B\left(r\right)=L\left(r,r_{q}\right)\cdot q, \end{array}$$ (17)

where ***q*** = *q****d***_q_ denotes a current dipole with magnitude *q* pointing at direction ***d***_q_.

For simplicity, the orientation of the current dipole is assumed to be perpendicular to the surface. When taking measurements using the SARA device, we cannot obtain all the components of the magnetic field; instead, we get a specific sensor-oriented component. Under these conditions, Eq (17) becomes

$$\begin{array}{c} b\left(r\right)=L\left(r,r^{\prime }\right)q, \end{array}$$ (18)

where *b* denotes the sensor-oriented magnetic field measurement that is generated by a normal current dipole with magnitude *q*. Note that according to Eq (18), the lead field $L(r,r^{\prime })$ is exactly the same as the magnetic field measurement *b* at *r* if we apply a unit normal current dipole with *q* = 1 at ***r***′. If the volume conductor is spherically symmetric and piecewise homogeneous, the lead field for the normal component of the magnetic field has a closed form [19]. However, it is a great challenge to obtain an explicit expression for the lead field in a complex volume conductor. We choose to solve this problem using FEM, which is a powerful tool for numerically solving for the lead field since it can deal with the anisotropy and realistic geometry in our volume conductor.

In this work, considering the random initiation area [13, 14], we adopt the distributed source current instead of a small number of current dipoles. In this case, a much larger number (usually greater than 5,000) of current dipoles are distributed over the whole myometrium. Since the myometrium is a thin layer [20], it is feasible for us to assume that the source current is limited to the external uterine surface. We divide the external uterine surface into small elements and introduce a current dipole at every vertex of the elements. Therefore, the lead field $L(r_{i},r_{j}^{\prime })$ is the numerical solution *b*(***r***_*i*_) of the sensor-oriented magnetic field at sensor location ***r***_*i*_ generated by a unit current dipole at $r_{j}^{\prime }$, i.e.,

$$\begin{array}{c} L\left(r_{i},r_{j}^{\prime }\right)=b\left(r_{i}\right)≜g\left(r_{i},r_{j}^{\prime }\right),\;i=1,2,\ldots ,M,\;j=1,2,\ldots ,N, \end{array}$$ (19)

where *M* is the number of SARA sensors and *N* denotes the number of current dipoles. The linear relationship between the current dipole amplitudes ***q***_*t*_ and the measurements ***b***_*t*_ at time *t*, therefore, is given by

$$\begin{array}{c} b_{t}=[\begin{array}{c} b_{t}\left(r_{1}\right)\\ \vdots \\ b_{t}\left(r_{M}\right) \end{array}]=[\begin{array}{ccc} g(r_{1},r_{1}^{\prime }) & \cdots & g(r_{1},r_{N}^{\prime })\\ \vdots & \ddots & \vdots \\ g(r_{M},r_{1}^{\prime }) & \cdots & g(r_{M},r_{N}^{\prime }) \end{array}]\;[\begin{array}{c} q_{t}\left(r_{1}^{\prime }\right)\\ \vdots \\ q_{t}\left(r_{N}^{\prime }\right) \end{array}]=Gq_{t},\;t=1,2,\ldots ,T, \end{array}$$ (20)

where

1. *b*_*t*_(***r***_*i*_), *i* = 1, 2, …, *M* is the magnetic field measured by *i*th sensor at time *t*;
2. $q_{t}\left(r_{j}^{\prime }\right),\;j=1,2,\ldots ,N$ is the amplitude of the *j*th current dipole at time *t*;
3. $G=\{g\left(r_{i},r_{j}^{\prime }\right),\;i=1,2,\ldots ,M,\;j=1,2,\ldots ,N\}$ is the lead-field matrix, with $g(r_{i},r_{j}^{\prime })$ obtained using Eq (19).

### Inverse estimation of source currents

The data collected by all sensors at time *t* can be expressed as

$$\begin{array}{c} b_{t}=Gq_{t}+e_{t}, \end{array}$$ (21)

for *t* = 1, 2, …, *T*, where ***e***_*t*_ is the measurement noise at time *t*. With the constructed lead-field matrix ***G***, we are interested in estimating the current dipole amplitudes ***q***_*t*_ from the magnetic fields ***b***_*t*_ measured using the SARA device. In the MMG inverse estimation problem, the number of unknowns, *N*, is usually greater than 5,000, but the number of measurements, *M*, is about one hundred. Because of this ill-posed nature, the inverse problem has an identifiability issue, in that there is no unique mathematically correct solution for the problem. To resolve this issue, it is necessary to impose additional constrains on the current dipoles. Here, we are interested in the most significant source currents, hence we embed extra information as an *ℓ*_1_ norm on the current distribution [21]. The resulting convex optimization problem is known as the Lasso problem [22]:

$$\begin{array}{c} {\hat{q}}_{t}=\arg\min_{q_{t}}\frac{1}{2}\|b_{t}-Gq_{t}{\|}_{2}^{2}+\lambda {\left\|q_{t}\right\|}_{1},\;t=1,2,\ldots ,T, \end{array}$$ (22)

where λ is a regularization parameter balancing the least-squares error and the *ℓ*_1_ penalty. A small λ puts more emphasis on the least-squares error, whereas large values of λ emphasize the *ℓ*_1_ penalty. Applying convex optimization algorithms [23], we can obtain the source current amplitudes after solving this Lasso problem.

### Prediction of intrauterine pressure

During pregnancy, the uterine contractile activities appear in a form of an intrauterine pressure increase. We employ an absolute-value-based method [24] to predict the intrauterine pressure from our estimated source currents. To each estimated source current at each location, we first apply a 4th-order low-pass Butterworth filter with a cut-off frequency of 2 Hz and then downsample the source currents with a sampling frequency of 4 Hz to lower the computational complexity. After rectifying the source currents, we obtain the approximate energy by summing over all locations. We then smooth the energy by applying a 4th-order low-pass Butterworth filter with a cut-off frequency of 0.02 Hz and resample at the original sampling frequency, resulting in our predicted intrauterine pressure.

## Results

In this section, we first illustrate the constructed lead-field matrix and then present numerical examples using both synthetic and real MMG data to illustrate our approach.

For the construction of the lead-field matrix, we divided the external uterine surface into 12,412 vertices, i.e., *N* = 12,412. The sensor-oriented magnetic field is measured on the abdominal surface using the SARA device with 151 sensors, i.e., *M* = 151. The constructed lead-field matrix ***G*** is therefore a 151-by-12,412 matrix, corresponding to the SARA device with 151 sensors and 12,412 unit current dipoles on the external uterine surface.

### Validation of the constructed lead-field matrix

**Fig 3:**
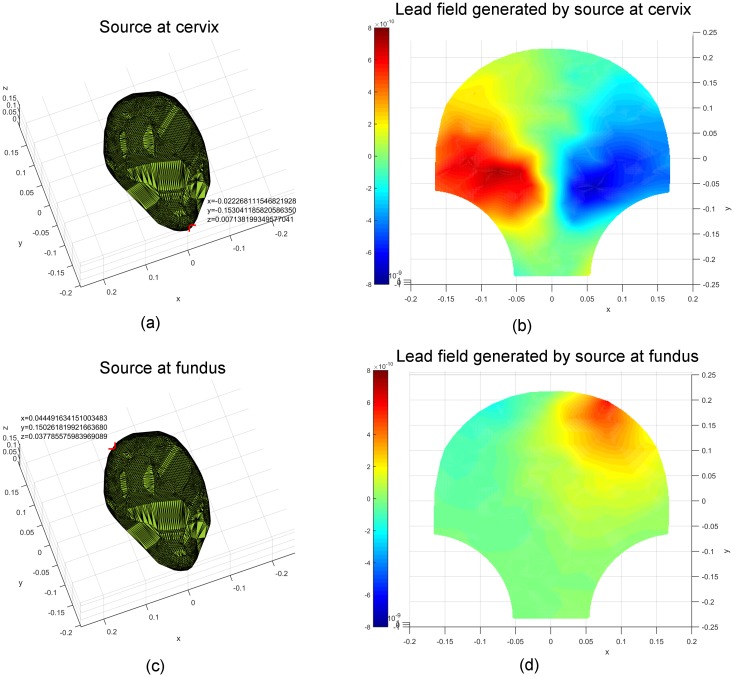
Lead fields corresponding to unit current dipoles (locations highlighted in red) at the cervix and fundus of the uterus. (a) A unit current dipole at the cervix (−0.022, −0.153, 0.007). (b) The corresponding lead field generated by the unit current dipole at the cervix. (c) A unit current dipole at the fundus (0.044, 0.150, 0.038). (d) The corresponding lead field generated by the unit current dipole at the fundus.

**Fig 4:**
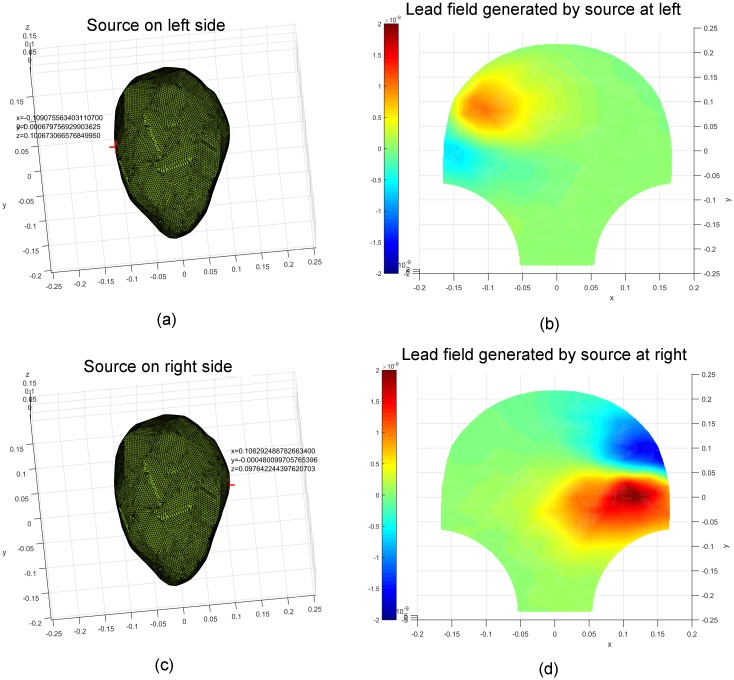
Lead fields corresponding to unit current dipoles (locations highlighted in red) on the left and right side of the uterus. (a) A unit current dipole on the left side (−0.109, 0.0007, 0.101). (b) The corresponding lead field generated by the unit current dipole on the left. (c) A unit current dipole on the right side (0.106, −0.0004, 0.098). (d) The corresponding lead field generated by the unit current dipole on the right.

**Fig 5:**
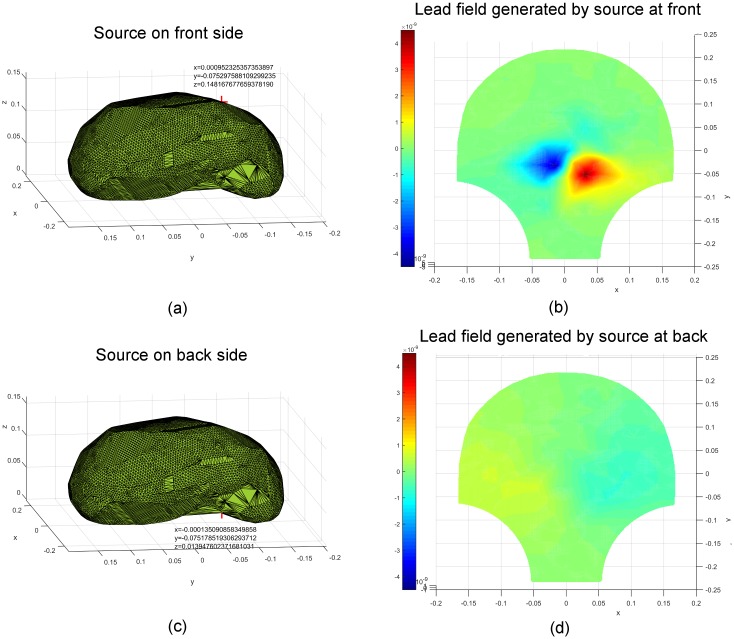
Lead fields corresponding to unit current dipoles (locations highlighted in red) on the front and back side of the uterus. (a) A unit current dipole on the front side (0.001, −0.075, 0.148). (b) The corresponding lead field generated by the unit current dipole on the front. (c) A unit current dipole on the back side (−0.0001, −0.075, 0.014). (d) The corresponding lead field generated by the unit current dipole on the back.

We displayed the lead fields corresponding to particular source locations on the SARA device to test the feasibility of the constructed lead-field matrix ***G***. In order to present the relationship between the lead field and source location and distance, the specific source locations were chosen to be pairs, such as cervix and fundus, left and right, front and back (from the front perspective). The corresponding results are illustrated in Figs 3–5. The values of the spatial coordinates (*x*, *y*, *z*) are expressed in meters. Figs 3 and 4 show the source coordinates at the cervix (−0.022, −0.153, 0.007), fundus (0.044, 0.150, 0.038), left (−0.109, 0.0007, 0.101), and right (0.106, −0.0004, 0.098) and the corresponding lead fields. We observe that the magnetic-field patterns appear at the corresponding surrounding areas of the sources. The lead fields generated by the sources at the front (0.001, −0.075, 0.148) and back (−0.0001, −0.075, 0.014) of the uterus are presented in Fig 5. Note that the magnetic-field intensity generated by a source at the front is higher than that generated by the one at the back, which is in agreement with the inverse square nature of magnetic field with respect to the distance between source and observation points.

### Estimation using synthetic MMG data

**Fig 6:**
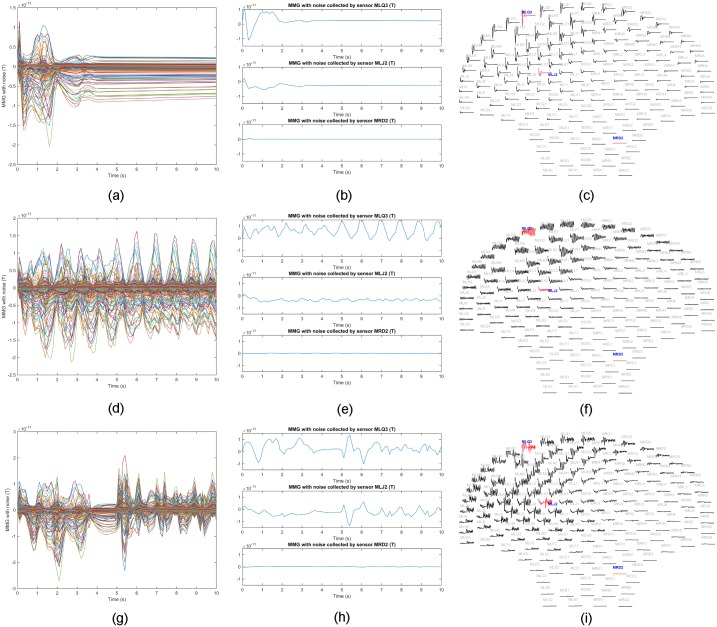
Sensor temporal courses of synthetic MMG data sets in Table 1. (a) Synthetic MMG over all sensors with each curve representing the temporal course over one sensor for Data Set 1. (b) Example traces for Data Set 1 from sensors MLQ3, MLJ2, and MRD2, respectively. (c) Layout plot of Data Set 1 over SARA device. (d) Synthetic MMG over all sensors with each curve representing the temporal course over one sensor for Data Set 2. (e) Example traces for Data Set 2 from sensors MLQ3, MLJ2, and MRD2, respectively. (f) Layout plot of Data Set 2 over SARA device. (g) Synthetic MMG over all sensors with each curve representing the temporal course over one sensor for Data Set 3. (h) Example traces for Data Set 3 from sensors MLQ3, MLJ2, and MRD2, respectively. (i) Layout plot of Data Set 3 over SARA device.

We utilized three synthetic MMG data sets (see details in Table 1 and Fig 6) to test our inverse estimation approach. Since fundus is one of the sites where uterine contractions are observed to arise [15–17], we excited the uterine activity at the upper left (from the rear perspective) of the uterus in the simulations. The parameters of the cell-level model in our multiscale model were set to generate plateau-type and bursting-type action potentials, which are the two predominant types in both a single uterine smooth muscle cell and isolated strips of myometrium [25, 26]. In each simulation, the sampling frequency was 10 Hz with simulation length of 10 s. We obtained the synthetic MMG data ***b***_*t*_, *t* = 1, 2, …, 100 as 100 151-by-1 vectors and the lead-field matrix ***G*** as a 151-by-12,412 matrix, i.e., *M* = 151, *N* = 12,412, and *T* = 100.

**Table 1:** Details of three synthetic MMG data sets.

Data set	Initiation area	Action potential	Sampling frequency	*M*	*N*	*T*
**1**	1	plateau	10 Hz	151	12,412	100
**2**	1	bursting	10 Hz	151	12,412	100
**3**	2	bursting	10 Hz	151	12,412	100

**Fig 7:**
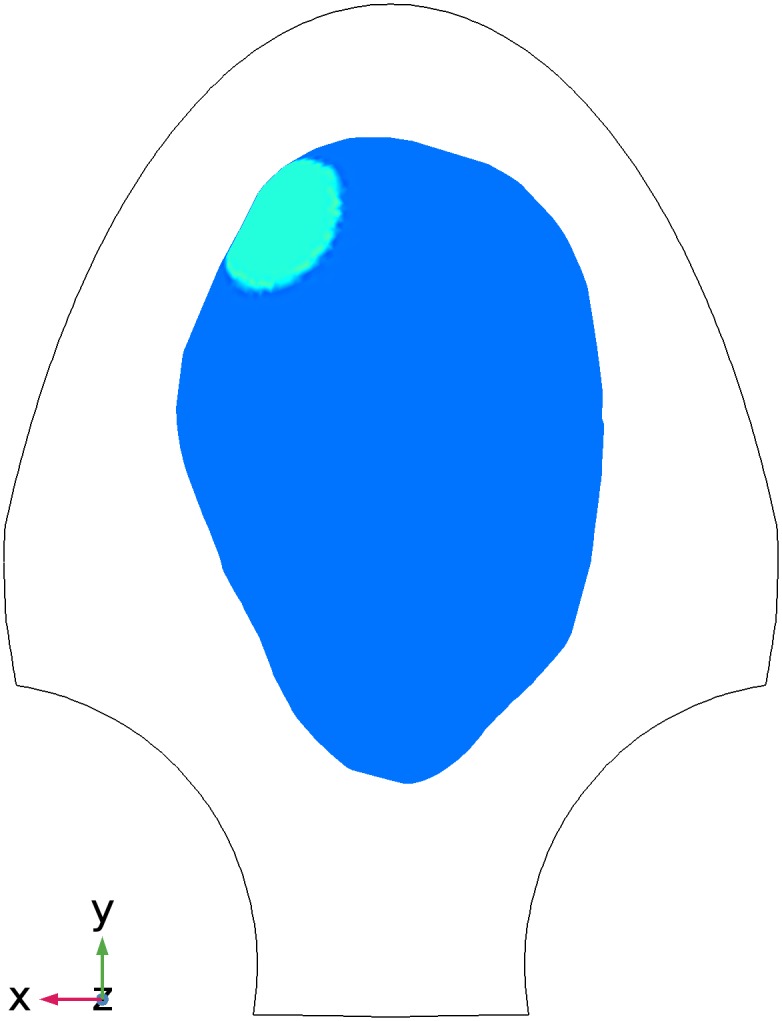
Location of the initiation area in the uterus for Data Sets 1 and 2 in Table 1. Blue, uterus; green, initiation area; black, the contour of SARA device.

**Fig 8:**
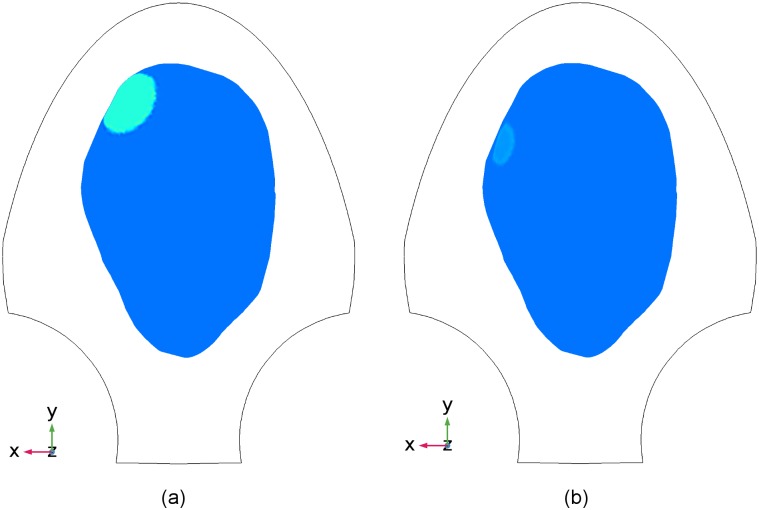
Locations of the initiation area in the uterus for Data Set 3 in Table 1. Blue, uterus; green, initiation areas; black, the contour of SARA device. (a) The first location. (b) The second location.

The initiation area of the first two synthetic MMG data sets is illustrated in Fig 7, in which the uterus is drawn in blue with the initiation area highlighted in green. The contour of the SARA device is sketched in black curves. The first data set represents the short-time oscillations of magnetic field (Fig 6a), that correspond to plateau-type action potential recruiting a small region in the upper left of the uterus (Fig 6c). In the second data set, local activity of bursting-type action potential (Fig 6f) was adopted to generate long-time oscillations of magnetic field (Fig 6d). Regarding the change of initiation areas during a single contraction or successive contractions [14], we shifted the initiation area from the location illustrated in Fig 8a to that in Fig 8b during one contraction, resulting in a local contractile activity shown in Fig 6i.

In the literature, there are many ways to define the regularization parameter λ [27]. In general, the degree of the regularization should be consistent with the level of noise involved in the measurements. Therefore, the regularization parameter λ was set to be a little bit greater than the noise in our estimation. The choice of the regularization parameter is beyond the scope of this work.

**Fig 9:**
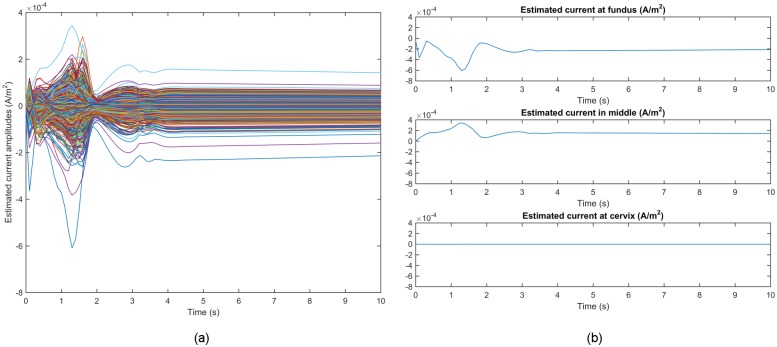
Temporal courses of estimated source current amplitudes for Data Set 1 in Table 1. (a) Estimated source current amplitudes at all source locations with each curve representing the temporal course over one source location. (b) Example traces of estimated source current amplitudes at the fundus, middle, and cervix of the uterus, respectively.

**Fig 10:**
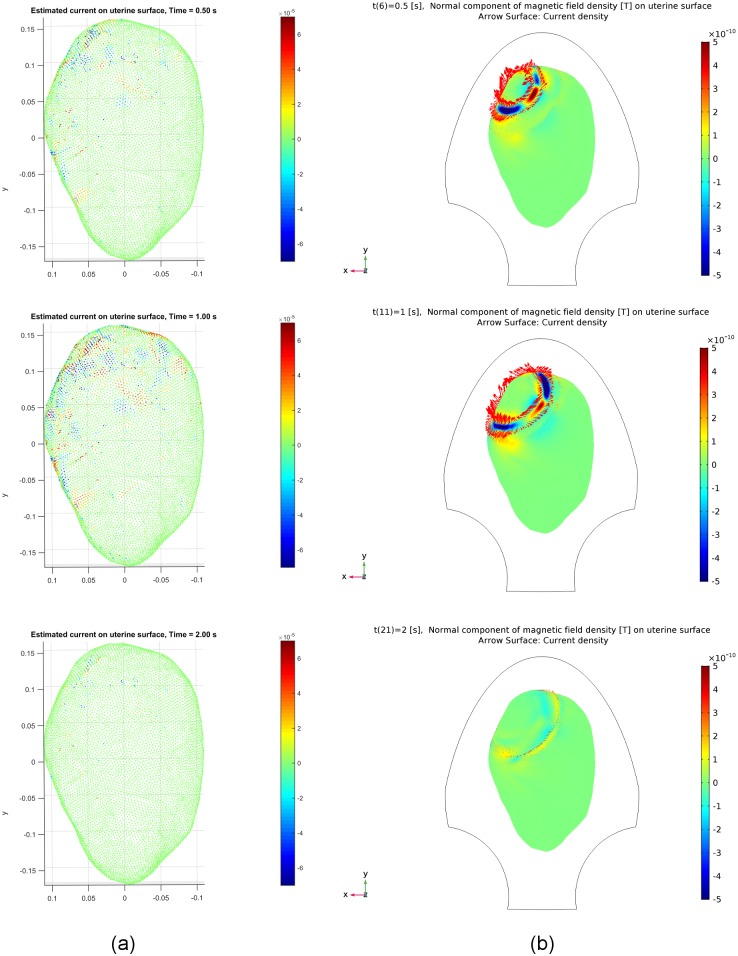
Estimated and synthetic source current amplitudes at time instants *t* = 0.5 [s], 1.0 [s], 2.0 [s] for Data Set 1 in Table 1. (a) Estimated source current amplitudes (see the corresponding video in S1 Video). (b) Synthetic source current amplitudes generated by our multiscale model.

Fig 9 shows the time courses of the estimated source currents for Data Set 1 in Table 1. Here, we were interested in the ones with higher intensity, hence set the threshold of the absolute value of amplitudes to be 2 × 10^−5^. Fig 10a illustrates snapshots of the estimated source currents on the uterine surface after thresholding at different time instants *t* = 0.5 s, 1.0 s, 2.0 s. The synthetic source currents generated using our multiscale model are presented as the Arrow Surface (red arrows on the uterine surface) in Fig 10b, in which the synthetic MMG on the uterine surface is also displayed. Red arrows in this figure reflect the direction of the source current. We observe that the synthetic source currents are distributed in a small local region in the upper left of the uterus at the beginning, then appear in a larger constrained neighboring region, and finally return to quiescence. Comparing Fig 10a with Fig 10b, we can see that the distribution area of the estimated source currents resembles that of the synthetic ones, although it is not exactly the same. Furthermore, while we did not consider the tangential component of the source current, the estimated source currents capture the emergence of local activities and the involvement of a larger excited area in the following contractile activities, which are in agreement with the tissue recruitment and contraction coordination via limited action potential propagation in local contractions.

**Fig 11:**
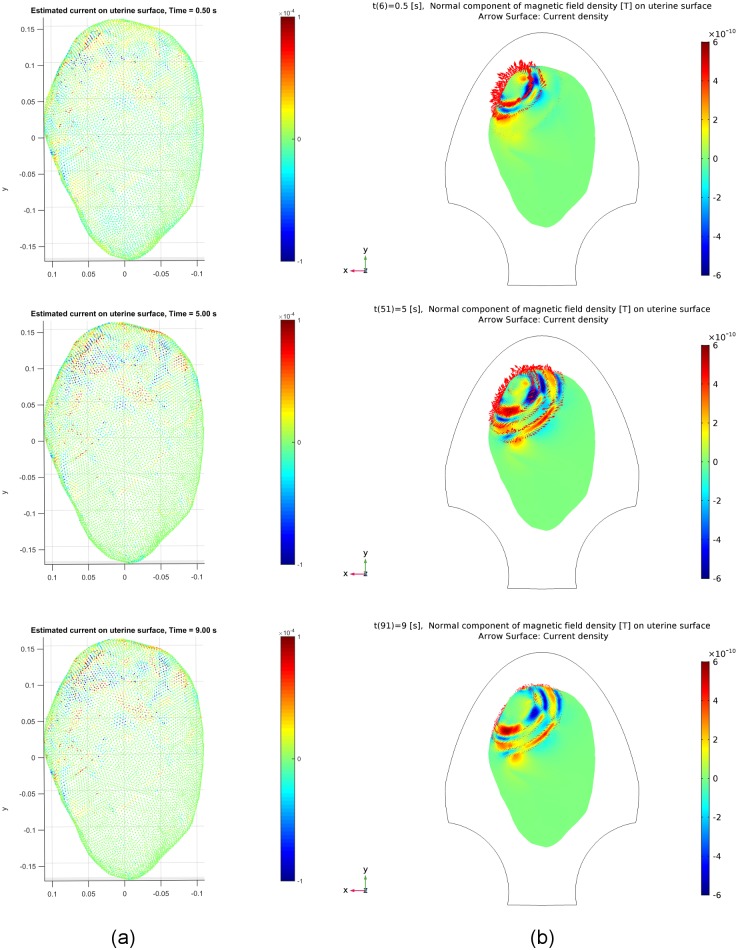
Estimated and synthetic source current amplitudes at time instants *t* = 0.5 [s], 5.0 [s], 9.0 [s] for Data Set 2 in Table 1. (a) Estimated source current amplitudes. (b) Synthetic source current amplitudes generated by our multiscale model.

**Fig 12:**
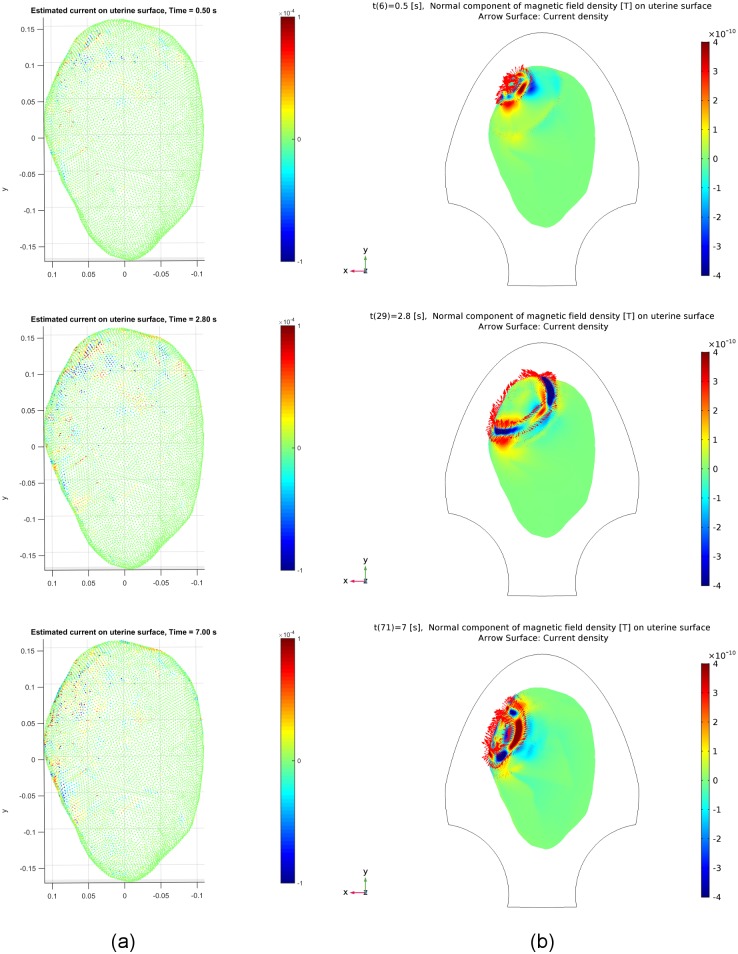
Estimated and synthetic source current amplitudes at time instants *t* = 0.5 [s], 2.8 [s], 7.0 [s] for Data Set 3 in Table 1. (a) Estimated source current amplitudes. (b) Synthetic source current amplitudes generated by our multiscale model.

The snapshots of the estimated source currents for Data Sets 2 and 3 in Table 1 are illustrated in Figs 11 and 12, respectively. We observe that similar results are obtained for both data sets, i.e., the occurrence of local activity and recruitment of neighboring area for the synthetic and estimated source currents are fairly well matched. Note from the results at different time instants, *t* = 2.8 s and *t* = 7.0 s, in Fig 12 that the estimated source currents reflect the change of the excited area from the upper left to a relatively lower position during the contraction, due to the initiation area change as shown in Fig 8.

### Estimation using real MMG data

**Fig 13:**
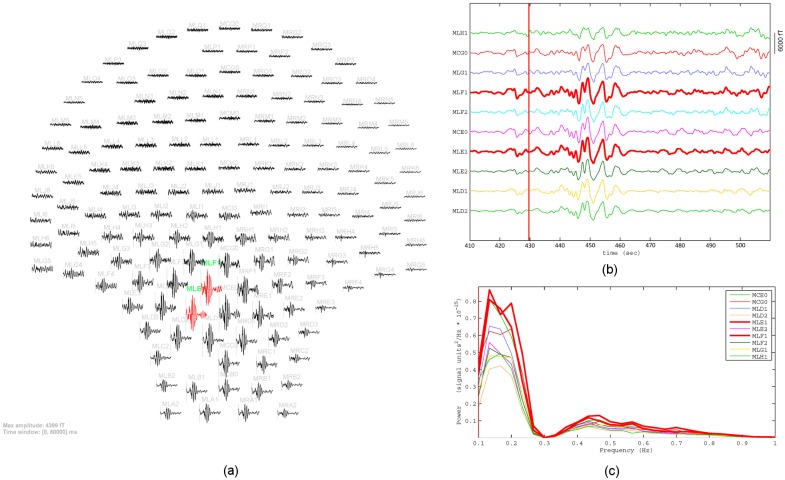
Preprocessed MMG signals of Data Set 2 in Table 2. (a) Layout plot of MMG signals over SARA device that covers one contraction (430 s − 490 s). (b) Expanded view of MMG signals that were obtained from 10 sensors in the lower left side of the abdomen. (c) Frequency spectrum obtained from these sensors.

We validated our inverse estimation method using three real data sets (see details in Table 2). Representative MMG signals after preprocessing that is described in subsection Clinical site and MMG data are illustrated in Fig 13. The MMG signals over the SARA sensors that covered one uterine contraction for 60 s are shown in Fig 13a. The signals from sensors MLE1 and MLF1 with high amplitudes are highlighted in red. We can see strong uterine activities in the lower left region of the abdomen. Fig 13b presents an expanded view of the signals obtained from 10 sensors in this region. The frequency spectrum in the frequency band of 0.1 − 1 Hz obtained from these sensors are given in Fig 13c.

**Table 2:** Details of three real MMG data sets.

Data set	Data	*M*	*N*	*T*
**1**	MMG & abdominal deflection	149	12,412	23,040
**2**	MMG	148	12,412	16,001
**3**	MMG	148	12,412	19,201

**Fig 14:**
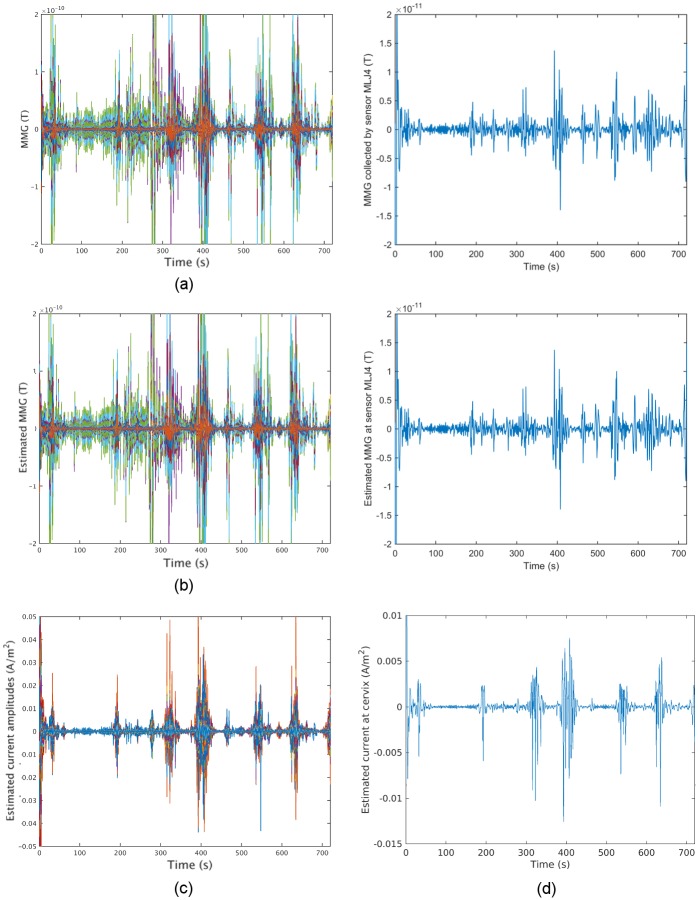
Sensor temporal courses of estimated source current amplitudes and the corresponding estimated MMG of Data Set 1 in Table 2. (a) Sensor temporal courses of real MMG data, with each curve representing the temporal course over one sensor. (b) Sensor temporal courses of the reconstructed MMG, using our estimated source current, with mean-squared error MSE = 7.50 × 10^−18^. (c) Temporal courses of estimated source current amplitudes, with each curve representing the temporal course over one source location. (d) Example traces of real MMG data, reconstructed MMG, and estimated source current amplitude, respectively.

**Fig 15:**
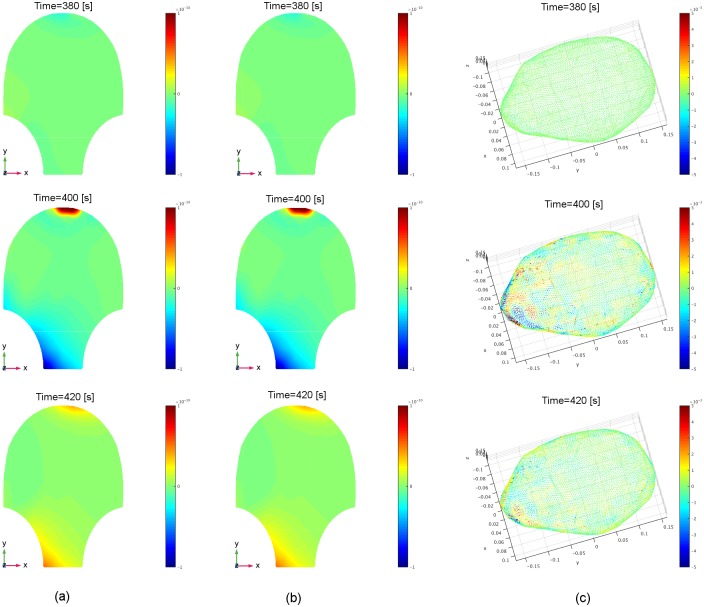
Estimated source current amplitudes and the corresponding estimated MMG of Data Set 1 in Table 2 at time instants *t* = 380 [s], 400 [s], 420 [s]. (a) Real MMG data on the abdominal surface collected by the SARA device (see the corresponding video in S2 Video). (b) Reconstructed MMG on the abdominal surface, using our estimated source currents. (c) Estimated source current amplitudes in the myometrium (see the corresponding video in S3 Video).

For the first MMG data set that lasts for 12 minutes, we removed MMG data from two noisy sensors and also removed two corresponding rows of the lead-field matrix, thus obtaining the MMG data ***b***_*t*_, *t* = 1, 2, …, 23, 040 as 23,040 149-by-1 vectors and the lead-field matrix ***G*** as a 149-by-12,412 matrix, i.e., *M* = 149, *N* = 12,412, and *T* = 23,040 (see Data Set 1 in Table 2). The temporal courses of the MMG data over 149 sensors are shown in Fig 14a, with amplitude in Tesla (T). The MMG data collected when the woman experienced no contractions is at the level of several 10^−13^s. Therefore, the regularization parameter λ was set to be 10^−12^, slightly greater than the noise, in this estimation. The estimated MMG and source currents are shown in parts b and c of Fig 14. As can be seen from Fig 14, the source-current patterns match well with the real MMG data, i.e., there are stronger source currents underlying more intense uterine electrical activities. In addition, we observe that the reconstructed MMG using our estimated source currents is in fair agreement with the real MMG data. Fig 15 shows several snapshots of the real MMG data and corresponding estimated MMG and source current amplitudes. Fig 15a illustrates the real MMG data on the abdominal surface, and Fig 15b shows the reconstructed MMG data on the abdominal surface using the estimated source currents in the myometrium in Fig 15c. According to the non-invasive MMG recording on the abdominal surface, the cervix is active during this uterine contraction period, i.e., this contractile activity is constrained in the lower left region of the uterus. The strong signals in the top are due to maternal motion rather than uterine contractile activity (see the noisy signals in the top of Subfigure a of S1 Fig). The estimated source currents capture this local contraction: the source currents first arise in a small region in the lower left part of the uterus, then appear in larger regions when the contraction becomes stronger, and finally reverse this process.

**Fig 16:**
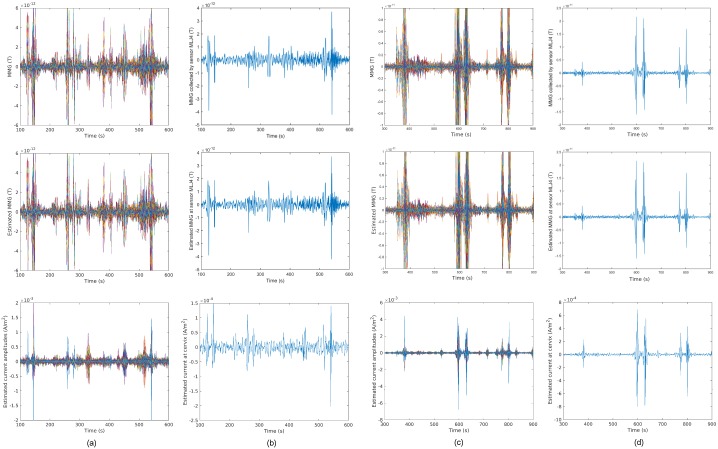
Sensor temporal courses of estimated source current amplitudes and the corresponding estimated MMG for Data Sets 2 and 3 in Table 2. (a) Data Set 2, with MSE = 1.51 × 10^−24^. (b) Example traces for Data Set 2. (c) Data Set 3, with MSE = 1.20 × 10^−23^. (d) Example traces for Data Set 3.

**Fig 17:**
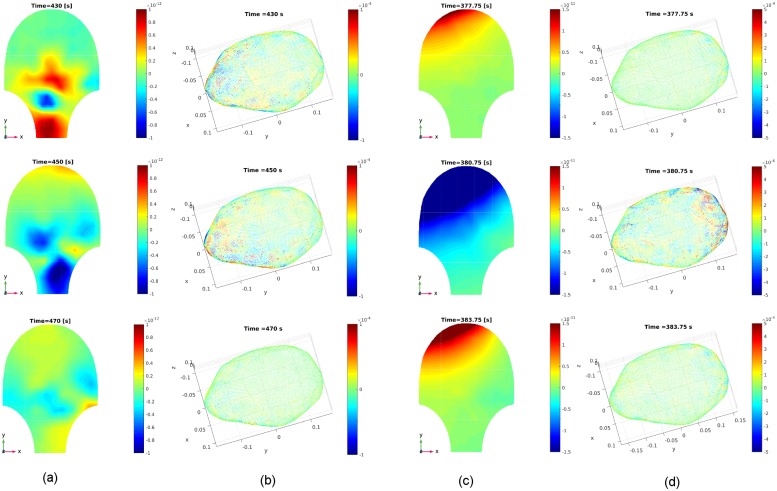
Estimated source current amplitudes for Data Sets 2 and 3 in Table 2 at different time instants. (a) Real MMG data for Data Set 2 at time instants *t* = 430 [s], 450 [s], 470 [s]. (b) Estimated source current amplitudes in the myometrium for Data Set 2. (c) Real MMG data for Data Set 3 at time instants *t* = 377.75 [s], 380.75 [s], 383.75 [s]. (d) Estimated source current amplitudes in the myometrium for Data Set 3.

For the other two data sets, we also removed the noisy sensors and the corresponding rows of the lead-field matrix before estimation (see Data Sets 2 and 3 in Table 2). In Figs 16 and 17, we illustrate the temporal courses and snapshots of the two MMG data sets and the estimated source currents, respectively. We observe that the results are similar to those of the first data set, i.e., the estimated source current density matches well with the local contraction in the lower region and upper left region of the uterus, respectively.

### Intrauterine pressure prediction

Currently, intrauterine pressure catheter or tocodynamometer is widely used by physicians for assessing uterine contractions during pregnancy. To show the potential clinical implications of our source current estimation despite the lack of ground truth, we predicted intrauterine pressure for Data Set 1 in Table 2, which includes simultaneous measurement of abdominal deflection during a contraction as described earlier.

**Fig 18:**
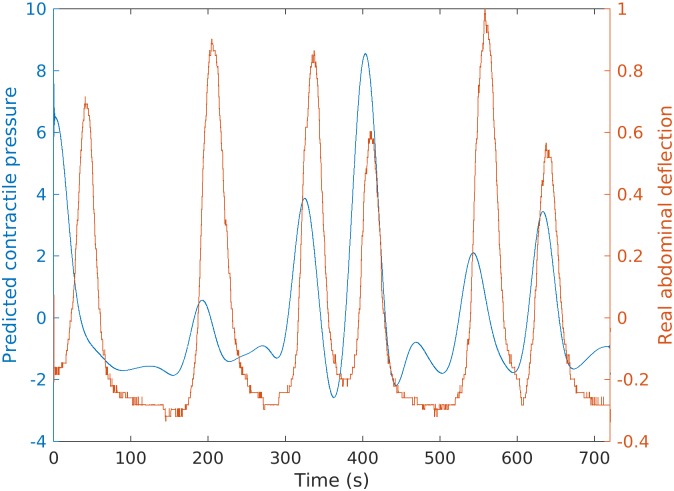
Real abdominal deflection observed during a contraction (red) and our predicted intrauterine pressure (blue) for Data Set 1 in Table 2.

**Fig 19:**
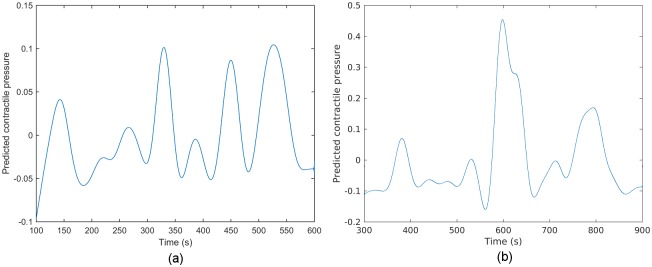
Predicted intrauterine pressure for Data Sets 2 and 3 in Table 2. (a) Data Set 2. (b) Data Set 3.

Fig 18 shows the real abdominal deflection data in red and our predicted intrauterine pressure in blue. We observe that 83.33% of the predicted intrauterine pressure peaks display good predictive timing of uterine contractions when compared with the abdominal deflection peaks. Note that the intrauterine pressure predicted from the myometrial source currents is several seconds in advance of the measured abdominal deflection, which is in agreement with the fact that the uterine electrical activities induce the increase of intrauterine pressure. According to the estimated source currents, there exist local contractile activities in uterus. Based on the mechanotransduction mechanism proposed in [28], a local contraction slightly increases the intrauterine pressure, resulting in a global wall tension increase and the induction of more local contractions that generate high intrauterine pressure. One possible reason for the difference of phase shifts between the real and predicted contractions is that the predicted contractions are calculated based on the local activities while the real ones are for the global change. The phase shift is dependent on the complex tissue recruitment and contraction coordination following the local contraction. The predicted intrauterine pressure curves for Data Sets 2 and 3 in Table 2 are also presented in Fig 19.

## Discussion

In this work, we tested our inverse estimation approach using synthetic MMG data sets which were generated using our previously developed multiscale model. The initiation areas were set at the fundus of the uterus (Fig 7) and the resulting electrophysiological activity further recruits more area of the uterus in local contractions (Figs 10 and 11). In real high-resolution recordings, there exist complex wave-propagation patterns, such as three or more wavefronts emerging at different positions of uterus and time instants and propagating in different directions [29]. According to our previous work [9], it is possible to obtain various magnetic-field patterns by changing the model configuration, such as initiation location, initiation time, and fiber orientations, which will affect the emerging position, emerging time, and propagating direction of the waves. MMG measurement is independent of any kind of reference, thus ensuring that the SARA device measures localized activity. The magnetic sensors of the SARA device are spaced 3 *cm* apart. Activities at different positions should be differentiable if the distance between them is greater than the distance between neighboring sensors. Our estimation result in Fig 12 reflects the shift of excited areas in uterus during one local contraction.

In our multiscale model, the coordination of uterine contractions is through the continuous propagation of action potential in smooth muscle cells. However, animal and human data indicates that action potentials propagate noncontiguously and over short distances [14, 30, 31]. We observe from the local contractions in real MMG data that the estimated source currents arise not only continuously, but also in areas that are not the neighbor of initiation area (Figs 15 and 17). One possible explanation for this is the mechanotransduction for long-distance signaling mechanism presented in [28]. In particular, one local contraction increases intrauterine pressure, then increases wall tension, inducing more local contractions to generate strong uterine contractile activity.

Uterine activities are generated by myometrial source currents [31]. It is common to model these currents using current dipoles [32]. According to the superposition principle, all complex sources can be approximated by multiple current dipoles. In this work, we are interested in the distribution of source currents and hence assume for simplicity that the current dipoles are perpendicular to the surface of the myometrium. Our numerical results (Figs 10–12, 15 and 17) show that the distribution of the source current estimation over the myometrium is in agreement with the synthetic source current and the MMG pattern measured using the SARA device, respectively. The snapshots of estimation results for synthetic MMG data (Figs 10–12) illustrate that our approach can track the emergence of local activity and the recruitment of larger area of source currents, although we do not consider the tangential component. Estimating the directions of source currents can be accommodated using the same framework. In this case, the inverse solution would be the amplitudes of the source currents in three orthogonal directions at each vertex, calculated using the lead-field matrix, in which each column corresponds to the lead field generated by a unit current dipole pointed in each direction.

Regarding the volume conductor, a set of concentric spheres with homogeneous and isotropic conductivities is the simplest model, in which case the radial component of magnetic field has a closed form with respect to a tangential current dipole [7, 19]. A variant on the spherical model introduced in our previous work [33] is a set of spheres with the outer uterine layer shifted to the front of the abdomen. In [34], a more complex volume conductor represented by differently shaped ellipsoids is developed, which was constructed based on anatomic diagrams from Hunter’s Anatomia Uteri Humani Gravidi [35]. In this project, we applied a more realistic volume conductor, proposed in our previous work [9], based on the MRI of a near-term woman and the abdomen that deforms to follow the SARA contour.

The spherical representation of the volume conductor geometry is a good approximation at the early stage of pregnancy, and its lead field can be expressed in a closed form. Our lead-field matrix, however, is constructed for a realistic geometry that is obtained after the acquisition of anatomical magnetic resonance images of a pregnant, near-term woman. In general, the computation of the lead field for this realistic geometry requires numerical solutions, for which we applied the finite element method, considering the anisotropic property of the myometrium. Numerical solution is computationally expensive and requires specifying conductivity values for each compartment. The conductivity values of the intracellular and extracellular domains, unlike those of the abdomen, amniotic fluid, and fetus [36], have not been reported. In this work, these values were obtained from the effective myometrium conductivity calculated by applying Archie’s law [37]. Regardless, construction of a lead-field matrix by using precise experimentally-confirmed conductivity values remains desirable.

The performance of the inverse calculation is sensitive to the regularization parameter λ. In general, the degree of the regularization should be consistent with the level of noise in the measurement data. Accordingly, the choice of λ is often determined by popular methods such as the discrepancy principle, *χ*^2^ test, L-curve, and generalized cross validation [27]. In this work, a fixed value of λ, determined according to the noise, was used for the estimation. Although we did not choose an optimal value, λ was set to ensure that the signal to noise ratios (SNRs) of the MMG data sets were in a consistent range in order to mitigate their potential influence. We postulate that optimizing the regularization parameter will further improve the performance of our estimation, the next natural research topic.

Source estimation has wide application in many different anatomical domains, such as the brain, heart, and uterus [5, 38–41]. The inverse problem is to estimate a large number of current dipoles from about one hundred measurements, which is mathematically ill-conditioned in the sense that various source configurations can produce the same magnetic field pattern. To solve it, it is necessary to impose additional constrains on the current dipoles. Among them, the most commonly used is the minimum-norm constraint, which imposes a weighted *ℓ*_2_ norm on the source current distribution [19, 42–44]. A nonlinear smooth constraint is included using Bayesian methods [45], whose performance depends greatly on the choice of prior distributions. In spite of these methods, it is quite difficult to validate the estimation accuracy. However, our first attempt at source estimation for uterine contractions, despite its limitations, is promising. Future research on developing a method to solve this inverse estimation is needed to achieve good estimation performance.

## Conclusions

We proposed a method to estimate the underlying source currents in the myometrium from noninvasive abdominal MMG measurements of uterine contractile activities during pregnancy. Our method incorporates electrophysiological and anatomical knowledge of uterine contractions. We developed a forward model to describe the relationship between the abdominal magnetic field and myometrial source currents based on a lead-field matrix and used this model to compute the unknown source currents in the myometrium. We introduced a realistic four-compartment geometry as the volume conductor model and a current dipole as the source model. We also applied the finite element method to construct the lead-field matrix. We obtained the estimation of underlying source currents in the myometrium by solving a constrained optimization problem. We also predicted the intrauterine pressure, which is clinically used for uterine contraction measurements, from the estimated source currents based on an absolute-value-based method. Finally, we displayed the lead fields that are generated by unit current dipoles at particular locations and illustrated our approach through numerical examples using both synthetic and real MMG data. We then estimated source currents and predicted the intrauterine pressure to show its clinical implications. Our work is potentially important as a starting point for helping characterize underlying activities of uterine contractions during pregnancy and future diagnosis of contractile dysfunction.

## Supporting information

S1 Dataset Raw signals for real MMG Data Set 1. (MAT)

S2 Dataset Raw signals for real MMG Data Set 2. (MAT)

S3 Dataset Raw signals for real MMG Data Set 3. (MAT)

S1 Fig Preprocessed signals of real Data Set 1. (a) Layout plot of MMG signals over SARA device. (b) MMG signals that were obtained from sensor MRA1 in the lower right side of the abdomen. (c) Simultaneous abdominal deflection measurement.
(TIF)

S2 Fig Preprocessed MMG signals of real Data Set 3. (a) Layout plot of MMG signals over SARA device from 580 s to 640 s. (b) Expanded view of MMG signals that were obtained from 10 sensors in the upper right side of the abdomen. (c) Frequency spectrum obtained from these sensors.
(TIF)

S1 Video Estimated source current on uterine surface for synthetic MMG Data Set 1. (AVI)

S2 Video Real MMG Data Set 1 measured using SARA device. (AVI)

S3 Video Estimated source current on uterine surface for real MMG Data Set 1. (AVI)
