# Supplementary figures and images for: Estimating uterine source current during contractions using magnetomyography measurements
Source: PLoS One. 2018 Aug 23;13(8):e0202184. doi: 10.1371/journal.pone.0202184 (PMC6121809; doi:10.1371/journal.pone.0202184)

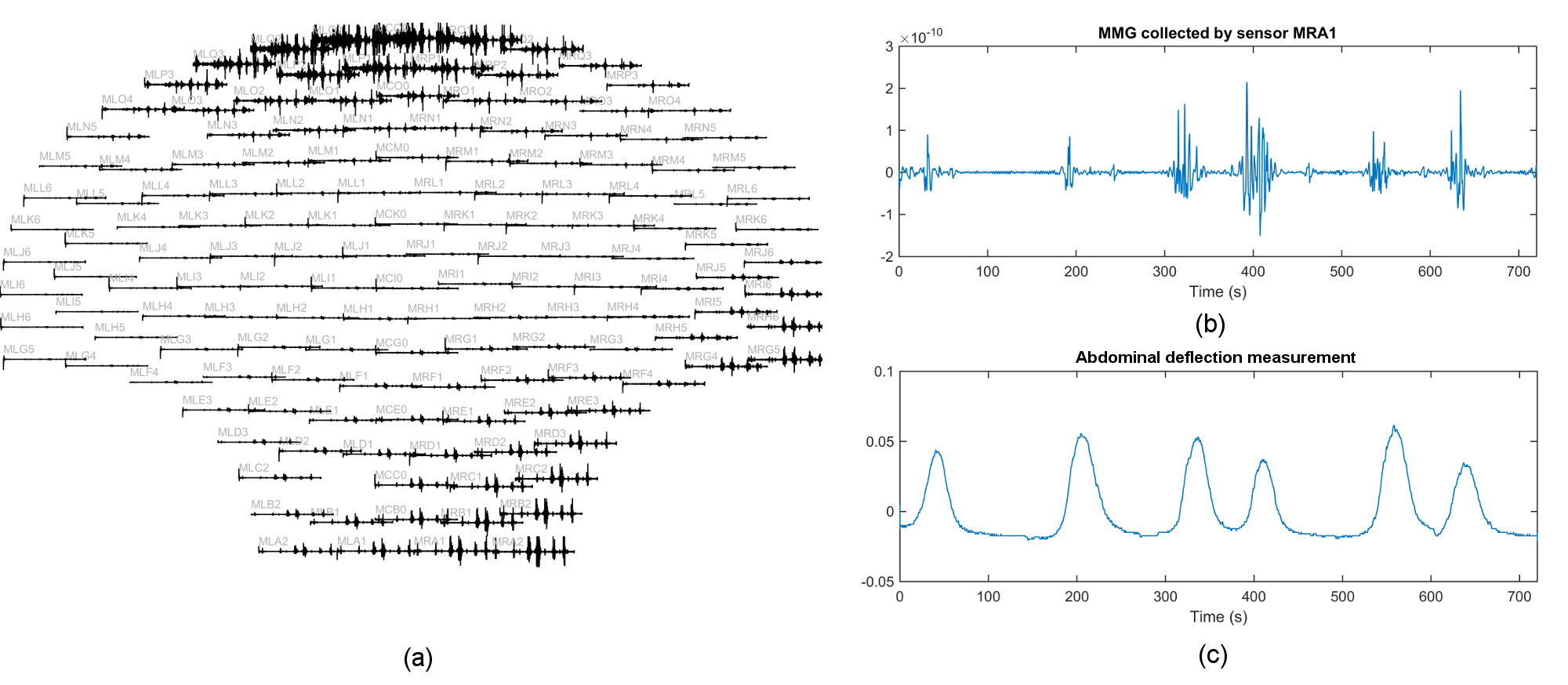

Supplement: S1 Fig — (a) Layout plot of MMG signals over SARA device. (b) MMG signals that were obtained from sensor MRA1 in the lower right side of the abdomen. (c) Simultaneous abdominal deflection measurement. (TIF) [file pone.0202184.s004.tif]

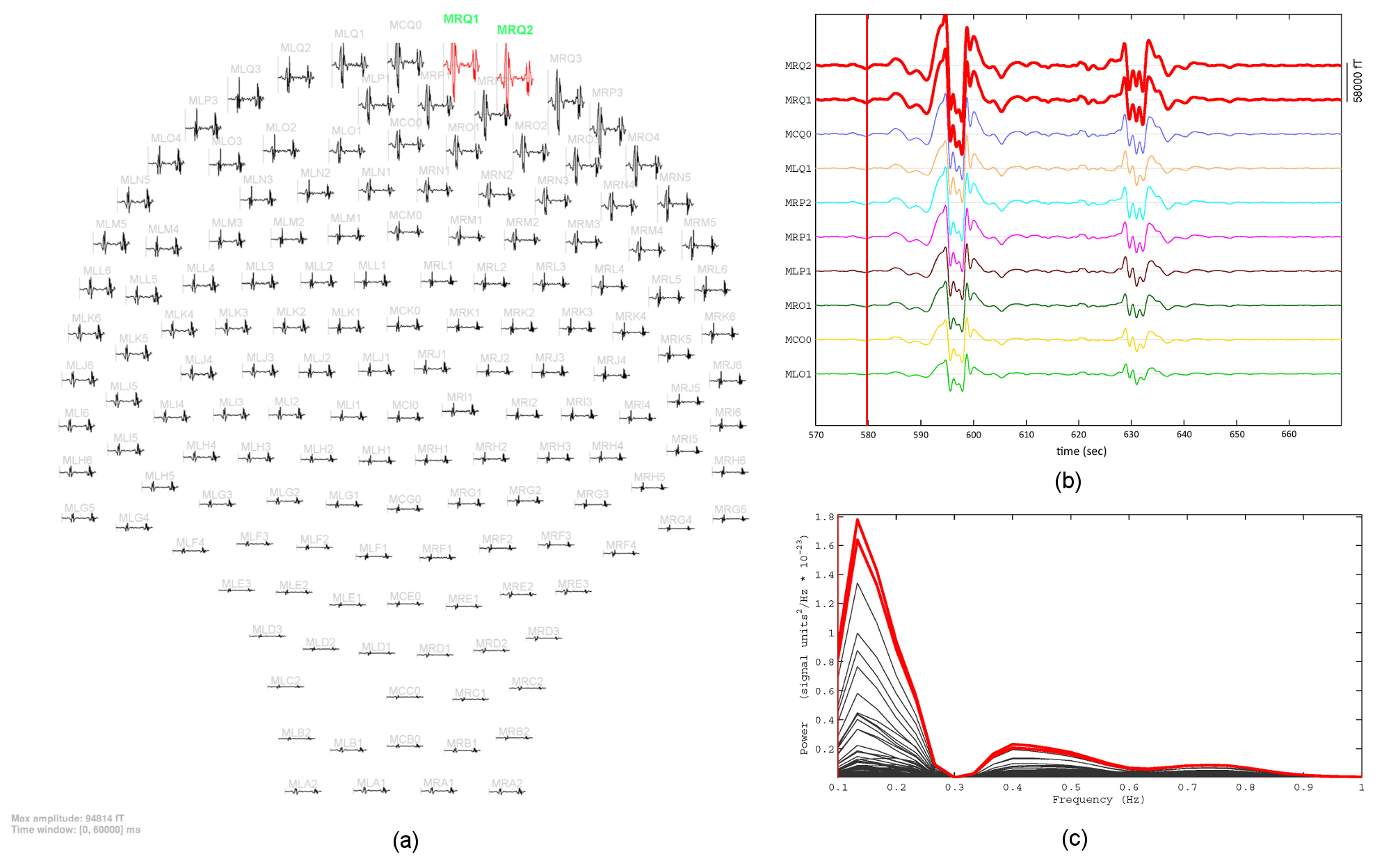

Supplement: S2 Fig — (a) Layout plot of MMG signals over SARA device from 580 s to 640 s. (b) Expanded view of MMG signals that were obtained from 10 sensors in the upper right side of the abdomen. (c) Frequency spectrum obtained from these sensors. (TIF) [file pone.0202184.s005.tif]
